# Water-Mellon Seed as a Dieuretic

**Published:** 1851-01

**Authors:** 


*Water Mellon Seed as a Dieuretic.*—In the November of the Charleston Medical Journal, Dr. Hook, of St. Matthews, S. C., bears strong testimony to the value of the seed of the water-mellon as a dieuretic, and gives a very interesting case illustrative of its powers. The editors of the Charleston Journal join their testimony with that of Dr. Hook, and we are able to corroborate their favorable opinion. Dr. Hook recommends that two ounces of the seed be bruised and a pint of boiling water poured over them. After cooling, one gill of this is taken at a dose, and in this way, is not only a demulcent, but an excellent dieuretic.

But we can assure Dr. Hook that we have seen much finer dieuretic effects from the formula we subjoin, than from any other dieuretic we have ever used. It has often succeeded when all others failed. In 1838 we reported in the predecessor of this Journal, a very remarkable case of suffering in the kidneys and bladder, in which the calls to urinate were almost incessant for two days and nights, and only one or two drops of urine could be passed at a time. The pain complained of resembled that described as an attendant upon stone in the bladder. Hip bathing, purgatives, emetics, opiates, and the usual round of dieuretics failed to give any relief. The patient seemed to be sinking rapidly under the combined efforts of pain, agitation, vigilance, and exhaustion. The anti-lithic paste was then resorted to for the first time, by the writer, and in less than half an hour after it was given the patient was easy, and slept for several hours. The kidneys acted freely, and all suffering ceased. Since that time abundant opportunities have presented themselves for the use of this paste, and its effects are uniformly all that the physician and patient can desire.

The formula for this paste was taught by Professor

John E. Cooke, and he gave strong testimony to its value. The following is the recipe:

| | | |
|---|---|---|
| ℞. | Castile soap, | ℥iv. |
| | Spermacetti, | ℥viij. |
| | Ven. Turpentine, | ʒvi. |
| | Ol. Anniseed, | ʒiij. |
| | Turmeric, | ʒij. |
| | Honey, q. s. | |

Rub the soap and spermacetti well together, then add the turmeric; after rubbing them well add turpentine and ol. anniseed; and sweeten with honey.

Of this paste, a piece the size of a nutmeg is given two or three times a day. The diseases in which it is most useful are those in which the mucous membrane is involved. There is a species of hoarseness which follows inflammatory action, and which often approaches aphonia, in which this paste is a very valuable remedy. B.

